# A Tale of Two Stressors in Biologic Drug Product Development: Shaking Mode and Primary Packaging

**DOI:** 10.1007/s11095-025-03959-4

**Published:** 2025-11-18

**Authors:** Siddhanth Hejmady, Elham Taherian, Reza Nejadnik

**Affiliations:** Department of Pharmaceutical Sciences and Experimental Therapeutics, 446, College of Pharmacy Building (CPB), University of Iowa, 180 S. Grand Ave, Iowa City, 52242 IA USA

**Keywords:** agitation, monoclonal antibody formulation, protein aggregation, surface adsorption, triple interface

## Abstract

**Purpose:**

Mechanical, interfacial, and shear stresses encountered during development, manufacturing and transportation of biologics can compromise monoclonal antibody (mAb) stability. However, most scale-down shaking models often depend solely on orbital agitation and overlook the effect of the solid–liquid interface. To study this gap, stress conditions were applied to simulate early-stage product development and real-world transportation in this work.

**Methodology:**

Accordingly, the aggregation profiles of Cetuximab and Tocilizumab formulations, with and without polysorbate 80 (PS80), were systematically compared after applying horizontal and orbital shaking. Protein aggregation was assessed using orthogonal techniques such as size-exclusion chromatography, dynamic light scattering, flow imaging microscopy, ultraviolet–visible spectroscopy, and visual inspection.

**Results:**

Horizontal shaking more effectively revealed Cetuximab’s susceptibility to aggregation under mechanical and interfacial stress whereas orbital shaking conditions were not as discriminative. Furthermore, to explore the effect of vial surface chemistry on subsequent protein aggregation, Cetuximab was subjected to horizontal shaking stress using both untreated and silanized glass vials. Interestingly, hydrophobic silanized vials without surfactant resulted in increased Cetuximab aggregation compared to untreated vials. In contrast, Cetuximab with PS80 showed fewer aggregates in silanized vials than in glass vials.

**Conclusion:**

These results underscore the value of selecting right-for-purpose agitation models and highlight the need to explore the triple interface for improving stress screening in drug product development.

**Supplementary Information:**

The online version contains supplementary material available at 10.1007/s11095-025-03959-4.

## Introduction

Biologic drug products encompass a range of therapeutic proteins and modalities, including monoclonal antibodies (mAbs), bispecific monoclonal antibodies (BsAbs), fusion proteins (FPs), antibody–drug conjugates (ADCs), and nanobodies. These biologics have made significant advances in the management and treatment of diseases such as rheumatoid arthritis, psoriasis, infectious diseases, and cancers. In 2024, protein-based therapies accounted for 32% of new drug approvals including 3 BsAbs, 2 FPs, and 10 mAbs, emphasizing their continual scientific and clinical relevance [1].

As a result, there is a constant interest in the enhancing the stability of mAb formulations in the biotechnological and pharmaceutical industries. Protein aggregation is a critical stability challenge in biologic drug product development, which can arise from numerous factors such as oxidation, heat, and interfacial stresses. Aggregates can pose significant issues by inducing anti-therapeutic antibodies and elevating immunogenicity risk. This can also contribute to decreased therapeutic efficacy, higher batch failures and production losses, and increased regulatory hurdles [2, 3]. Among the different stresses contributing to protein aggregation, interfacial stresses particularly occur at the multiple interfaces encountered throughout the lifecycle of a drug product including development, manufacturing, transportation, storage, and administration [4]. These interfaces include those of stainless steel vessels, syringe and vial walls, filter membranes, rubber stoppers, and even lubricating silicone oil interfaces, each with distinct molecular and biophysical properties that can influence protein behavior. Shaking-induced aggregation and particle formation is especially relevant during several stages, such as mixing and filtration during formulation, vibrations during transportation, and handling or transfer procedures during clinical administration [5]. For instance, agitation during shipping can mechanically disrupt interfacial films and viscoelastic gels formed by amphiphilic proteins molecularly adsorbed at interfaces, leading to their rupture and the release of aggregates into the solution [6, 7]. In this regard, Morales, and colleagues, representing the drug product handling working community across various pharmaceutical and biotechnological companies reported the use of scale-down models as proxies for real-world transport and handling to ensure quick screening and comparison of mAb formulations. Different shaking methods are among the most commonly employed methods and the formulation, type and intensity of shaking, container affect the generation of protein aggregates, specifically the formation of subvisible particles (SVP) [8].

A number of studies have shown that different shaking methods are effective in applying stress and have therefore used them to explore aggregation outcomes under specific conditions. Tawab *et al*. focused on evaluating particle formation in multiple mAb infusions prepared in intravenous (IV) bags subjected to vibrational orbital shaking with emphasis on the influence of clinical-use variables and formulation composition, for bevacizumab and pembrolizumab [9]. With respect to the effect of surfactant grade and its degradation on immunoglobulin G (IgG) formulations under various mechanical stresses, Grabarek and colleagues investigated the reduced functionality of polysorbate 80 (PS80) upon hydrolytic degradation in protecting IgG against aggregation [10]. Recent work by Fang *et al*. has even demonstrated the unexpected vulnerability of freeze-dried formulations over their liquid counterparts to shaking-induced degradation under vertical shaking, which emphasizes the importance of evaluating mechanical stress when choosing between liquid and freeze-dried formats [11]. Building on this, Jin *et al*. systematically optimized formulation variables by maintaining pH around 6.0, incorporating mannitol, and increasing protein concentration to mitigate significant physical degradation and aggregation under mechanical stress in freeze-dried mAb formulations [12]. Besides, Johann *et al*.'s work pioneered stress-controlled aggregation studies of diverse modalities including ADCs and bispecific FPs using microplates, and found that parameters such as higher shaking frequency, lower surface tension, and increased well volume-to-fill volume ratios distinctly increased interfacial stress and aggregation [13]. Similarly, Dasnoy and group addressed a distinct gap by demonstrating how shaker orbit settings and vial orientation, often overlooked variables, influence agitation-induced aggregation outcomes with small-orbit shaking inducing aggregation in vertical vials, and large-orbit shaking doing so in horizontal vials [14]. Also, previous research by Bai *et al*. used computational fluid dynamics (CFD) simulations for modeling stress intensities in common agitation setups, showcasing that systems such as vortex mixers generate high shear and surface turnover under idealized conditions [15]. Clearly, no standardized scale-down model for shaking currently exists, leading to variability in the parameters used by formulation scientists and product developers, complicating comparability of results across different laboratories. The majority of companies conducting shaking studies prefer orbital shakers, although reciprocating or horizontal, rotation and up and down shakers are also still used, as reported in a BioPhorum Development Group cross-company survey, in which 11 out of 13 respondents indicated this preference [16]. However, in spite of the diversity in available stress setups, experimental comparisons of mAb aggregation outcomes resulting from differential shaking modes, particularly horizontal versus orbital shaking, remain unexplored.

Furthermore, highlighting the gap in assessing the overlooked role of primary packaging material properties in mitigating stresses, Winter *et al*. demonstrated improved protein stability with polyvinylidene fluoride filters compared to cellulose acetate and polyethersulfone (PES). This was observed under simulated process stresses, underscoring the importance of selecting materials, filters or primary containers, that preserve protein integrity during handling [17]. There is evidence that aggregation outcomes under shaking are also influenced by interfacial chemistry and Wang *et al*. recently found how fluid shear-induced aggregates can be diminished utilizing hydrophobic surface-modified glass by multilayer adsorption of IgG2 during end-over-end rotation [18]. Additionally, Gerhardt *et al*. attributed particle generation in prefilled syringes to capillary forces disrupting protein layers at silicone oil–water-air interfaces during rotational agitation. This led to fragmented viscoelastic protein gels at the interface, dislodging aggregates and oil droplets into the bulk as mixed agglomerates [19]. Nevertheless, direct comparison of aggregation at solid–liquid-air interfaces across different vial surface chemistries, under real-world mechanical stress, remains an unexamined dimension of mAb stability and aggregation.

**Fig. 1:**
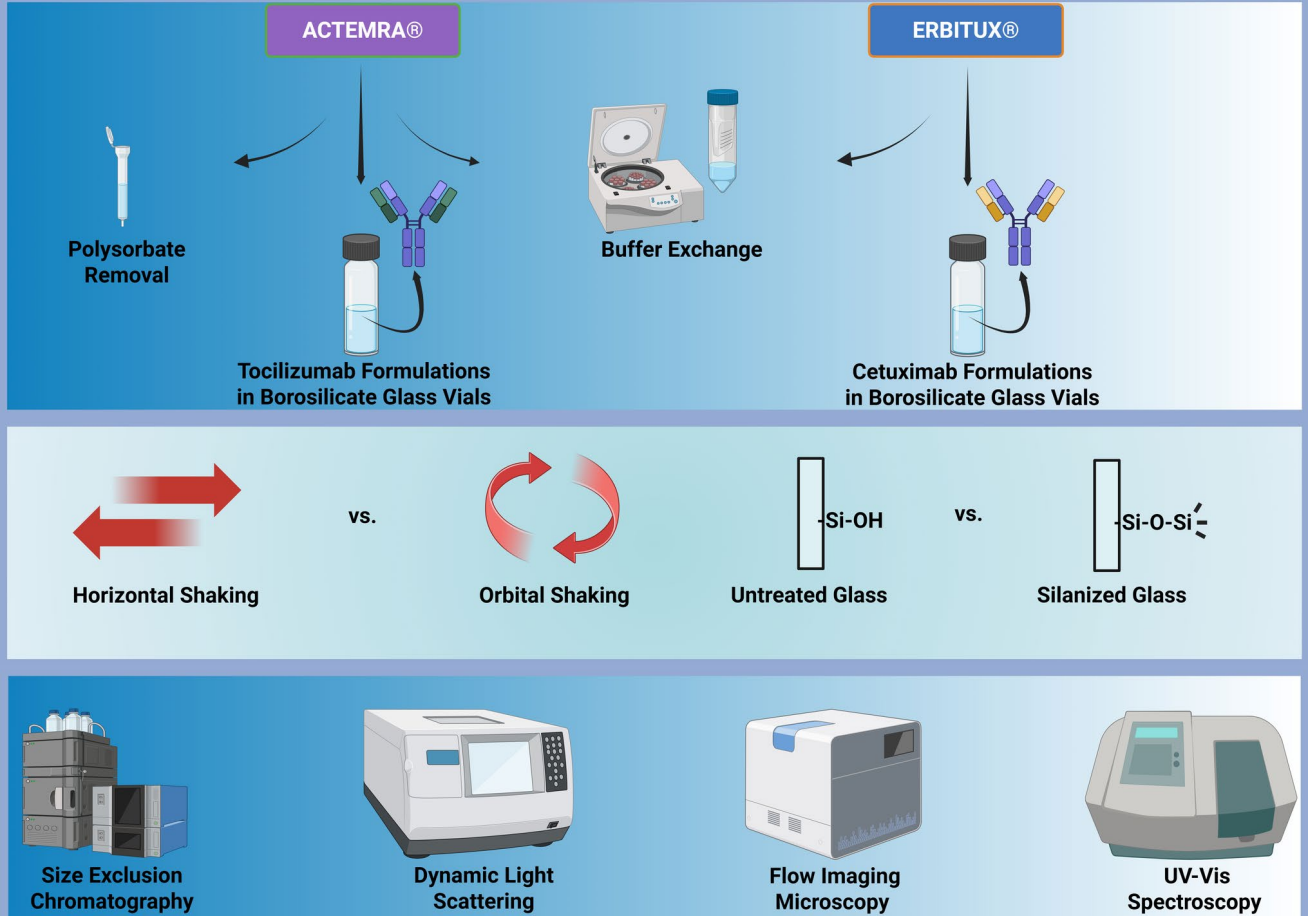
Schematic overview of the shaking stress study evaluating the impact of experimental parameters specifically the mode of shaking and primary packaging surface chemistry on mAb aggregation

Thus, the objective of this work is a systematic assessment of how differences in the experimental setup influence the aggregation outcomes of shaking stress experiments. The stressors under investigation include the mode of shaking, horizontal versus orbital, and the primary packaging, particularly the effect of vial surface chemistry and the resulting triple interface. The study examines the agitation-induced aggregation using Tocilizumab and Cetuximab, with Cetuximab further employed to specifically compare untreated and more hydrophobic silanized glass surfaces (Fig. 1).

## Materials and Methods

### Materials

The commercially available products, ERBITUX® (Cetuximab) and ACTEMRA® (Tocilizumab), were purchased and used as model antibodies in this study. PS80 was obtained from J.T. Baker, Phillipsburg, NJ, USA, and Dulbecco’s phosphate-buffered saline (1X, without calcium and magnesium) was obtained from Gibco Life Technologies, USA. Vivaspin® Turbo 15 Centrifugal Concentrators (30 kDa MWCO) were sourced from Sartorius, Goettingen, Germany, and Detergent-OUT™ Tween® was obtained from G-Biosciences, St. Louis, MO, USA. Trichloroethylene (TCE) was acquired from Thermo Fisher Scientific, Fair Lawn, NJ, USA and, dimethyldichlorosilane (DDS) was sourced from Sigma-Aldrich, Saint Louis, MO, USA. Hydrogen peroxide (30% solution), sulfuric acid, and methanol (HPLC grade) were obtained from Thermo Fisher Scientific, Fair Lawn, NJ, USA. 0.2 μm sterile PES filters were used for filtration, including Whatman™ membrane filters (Cytiva, Marlborough, MA, USA) and Nalgene™ Rapid-Flow™ filter units (Thermo Fisher Scientific, Waltham, MA, USA). Sodium phosphate monobasic monohydrate (NaH_2_PO_4_ • H_2_O) and sodium phosphate dibasic anhydrous (Na_2_HPO_4_) were obtained from Research Products International Corp, Illinois, USA. Multicompendial sodium chloride EMPROVE® ESSENTIAL was supplied by Merck KGaA, Darmstadt, Germany, and sodium azide was obtained from Thermo Fisher Scientific, Fair Lawn, NJ, USA. Ultrapure water (resistivity 18.2 MΩ·cm) from a Sartorius Arium® mini water purification system was used for the preparation of all samples.

### Surface Modification

**Fig. 2:**
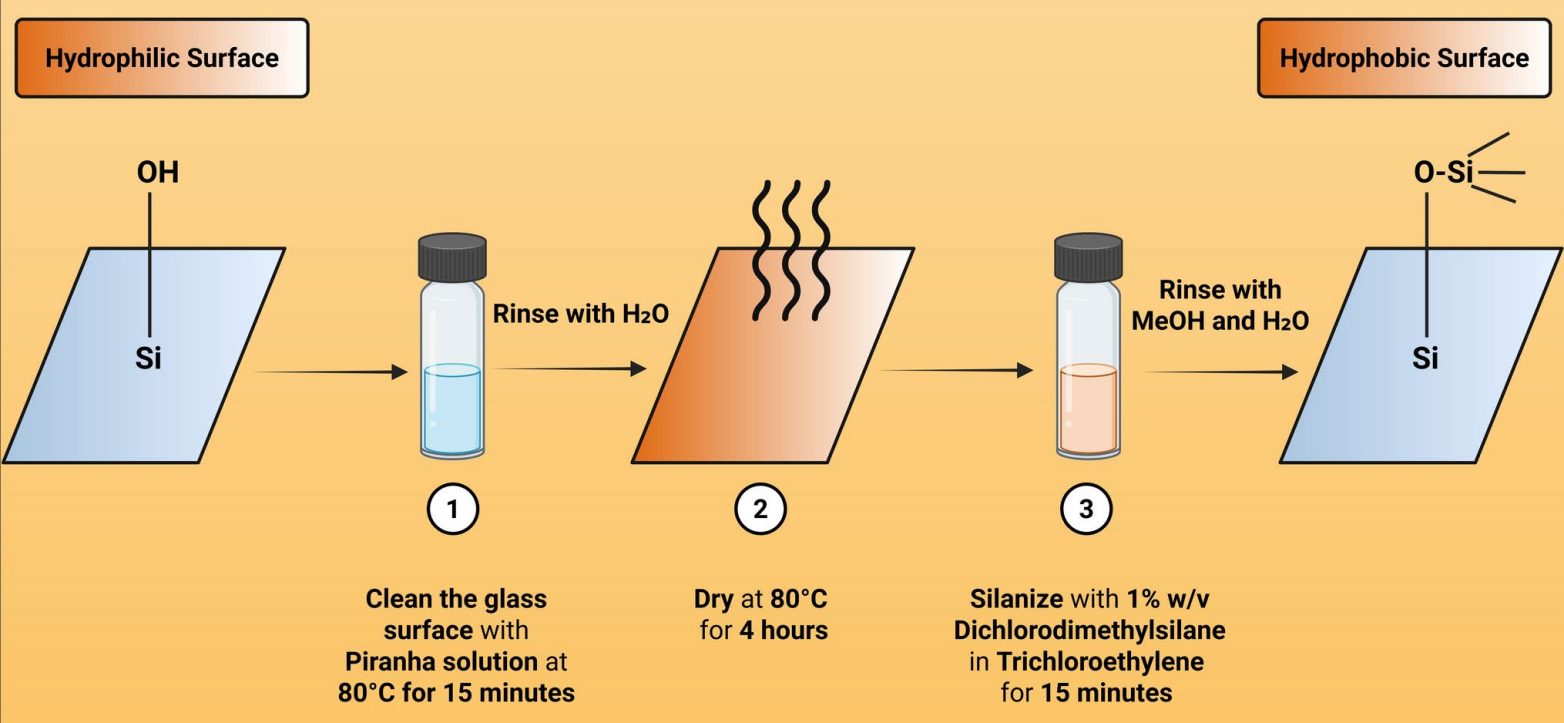
Silanization protocol for modifying glass surfaces by masking surface silanol groups with dimethyldichlorosilane (DDS), to render the surface hydrophobic

A surface modification protocol adapted from Nejadnik *et al*. was employed to silanize glass vial surfaces and mask Si–OH groups (Fig. 2) [20, 21]. 20.0 mL borosilicate glass scintillation vials (WHEATON®, DWK Life Sciences) were first cleaned using a 3:1 mixture of sulfuric acid and hydrogen peroxide at 80 °C for 15 min. They were then thoroughly rinsed with ultrapure water and kept immersed in ultrapure water. To produce a hydrophobic surface, the cleaned vials were dried at 80 °C for 4 h and then treated with 6.0 mL of a 1% w/v solution of DDS in TCE for 15 min. The DDS-coated vials were subsequently rinsed with methanol followed by ultrapure water and stored until use.

### Sample Preparation

Cetuximab was initially isolated from the marketed product ERBITUX®, and subsequently buffer-exchanged into PBS using centrifugal concentrators to prepare a stock solution at a concentration of 10.0 mg/mL. Tocilizumab was processed similarly, with PS80 removed from the marketed ACTEMRA® using detergent removal resin columns [22], followed by buffer exchange into PBS using centrifugal concentrators to obtain a 10.0 mg/mL stock solution. Both mAb solutions were filtered and diluted to 0.5 mg/mL in PBS. The formulations containing the surfactant PS80 were prepared with a concentration of 0.01% (w/v) PS80. Furthermore, the untreated glass vials were thoroughly rinsed with ultrapure water and dried prior to sample filling. Then, 2.0 mL of each 0.5 mg/mL prepared formulation (with or without PS80), was pipetted into each vial (either untreated or surface treated as described above) equipped with a screw cap closure and metal foil liner. All mAb sample preparations were carried out under laminar flow conditions and the protein concentrations were checked using a NanoDrop spectrophotometer (ThermoFisher Scientific, Waltham, Massachusetts, USA).

### Shaking Stress

To establish a sensitive and robust experimental set-up for evaluating agitation-induced aggregation, preliminary studies were conducted to optimize key parameters informing the final stress conditions utilized in this work [6]. Parameters evaluated included protein format (drug substance vs. drug product), primary container type and size, filling volume and concentration, headspace volume, shaking intensity, and duration. Based on these findings, shaking 2.0 mL fill volume of 0.5 mg/mL mAb in PBS at 200 RPM (rotations per minute) for 72 h in 20.0 mL vials was selected as the discriminatory condition for subsequent stress testing and comparative analyses of mAb formulations. All the vials were subjected to forced degradation studies at room temperature using two agitation modes: (1) orbital shaker (Digital Mini Rotators, Thermo Scientific™, Waltham, Massachusetts, USA) with a 10 mm orbit diameter, and (2) a horizontal shaker (SH-200 Series, Cole-Parmer®, Vernon Hills, Illinois, USA) with a 20 mm reciprocating stroke. For both the shakers, all the vials were placed in an upright position into tailor-made carton dividers to maintain vertical orientation and securely tightened to the shaker platforms to mimic real-life stresses (Fig. S1). Post-agitation, the T72 orbital and T72 horizontal samples were analyzed along with the buffer-only controls (with or without PS80), as well as unagitated controls, (1) T0 and (2) T72 Unshaken, which were stored under the same laboratory conditions as the agitated samples. All experimental conditions were performed in triplicate (*n* = 3).

### Visual Inspection

The vials containing the samples were assessed for visible particles immediately after removal from the shakers. All vials were swirled gently and slowly at a fixed viewing distance (~ 25 cm) for a consistent inspection time (~ 10 s per vial). Each sample was documented against both black and white backgrounds, and the observed particle number was semi-quantitatively scored using the following scale: (-) no visible particles, (+) few discrete visible particles, and (+ +) multiple or large visible particles. For triplicate measurements, the most frequent or consensus observation score among replicates was reported to differentiate the relative generation of visible particles.

### Size-Exclusion Chromatography (SEC)

An Agilent 1260 Infinity II HPLC system (Agilent, Santa Clara, California, USA) equipped with an AdvanceBio SEC column (200 Å, 4.6 × 300 mm, 1.9 μm) and a corresponding guard column was used to quantify the percentage of high molecular weight species (%HMWS) in the undiluted samples. The mobile phase consisting of 50 mM sodium phosphate buffer (pH 6.8) with 300 mM sodium chloride and 0.05% sodium azide, was filtered and degassed prior to usage. Chromatographic separation was performed at a flow rate of 0.2 mL/min under isocratic conditions, with 20 μL of sample injected per run into the system. Elution was monitored using a diode array detector at 220 nm and 280 nm for 25 min and the data were processed and analyzed using the chromatography data software, OpenLab.

### Dynamic Light Scattering (DLS)

DynaPro™ Plate Reader III (Wyatt Technology Inc., Santa Barbara, California, USA) was used to assess the average particle size in solution in the nanometer size range. All three replicate measurements were performed at a controlled temperature of 25 °C using 80 μL per well in a SensoPlate™ 96 well flat-bottomed microplates (Greiner Bio-One, Monroe, North Carolina, USA). A total of 10 acquisitions of the scattered light intensity fluctuations per sample, each with a 5-s acquisition time interval, were averaged using the software Dynamics 8.0.0.89. The decay of the scattered light intensity over time was analyzed by autocorrelation to derive the diffusion coefficient, which was used then as an input in the Stokes–Einstein equation to report the hydrodynamic diameter and polydispersity index (PDI).

### Flow Imaging Microscopy (FIM)

FlowCam 8000 (Yokogawa, Scarborough, Maine, USA) was used to perform FIM for evaluating the SVP count in the micrometer size range following stress. For each measurement, 500 μL of the undiluted sample was manually introduced into a 100 μm flow cell using a 10X objective lens, at a flow rate of 0.08 mL/min for real-time imaging. The flow cell was rinsed with ultrapure water between individual sample analyses, followed by purging the system with the corresponding buffer prior to the start of each run. The system output, reported in particles per mL, was determined for particles exceeding 2, 10, and 25 μm in diameter using the image analysis software, VisualSpreadsheet®.

### Ultraviolet–Visible (UV–Vis) Spectroscopy

Optical density at 350 nm (OD_350_) was measured using multi-mode plate readers (Molecular Devices, LLC, San Jose, California, USA) to quantify light-scattering indicative of the presence of protein aggregates. Spectra were collected from 200–450 nm for undiluted samples, and the scattering was assessed through OD_350_ measurements after baseline subtraction using the corresponding buffer signals.

### Statistical Data Analysis

All data are presented as mean ± standard deviation, unless otherwise stated. Statistical analyses, including two-way analysis of variance (2-way ANOVA) with Tukey’s multiple comparisons test were performed using Prism 10.3.1 (GraphPad Software, San Diego, CA, USA). The p-value indicated by * *p* < 0.05 was considered statistically significant.

## Results

All results presented below were generated under the mentioned shaking conditions, unless otherwise stated.

### Visible Particle Assessment by Visual Inspection

Visual inspection revealed noticeable differences in visible particle formation across various formulations as well as stress conditions (Table I and Fig. S2). At T0, all mAb samples were free from visible particles, with a score of (-), serving as baseline controls. After 72 h of horizontal shaking, Cetuximab without PS80 displayed the highest visible particle score of (+ +), observed primarily in both standard and silanized vials, with fibrous-like particles noted. The addition of PS80 appeared to mitigate this effect, reducing particle scores to (-) and (+) across Cetuximab formulations. In contrast, Tocilizumab generally showed lowest visible particle scores following stress, with (-) being the most common observation, regardless of PS80 presence and stress mode.

**Table I:** Visual inspection scores for visible particles in Cetuximab and Tocilizumab formulations under different shaking conditions

Monoclonal Antibody Formulation	Vial Type	T0	T72 Orbital	T72 Horizontal	T72 Unshaken
Cetuximab (− PS80)	Untreated	(-)	(-)	(+ +)	(-)
Tocilizumab (− PS80)	Untreated	(-)	(-)	(-)	(-)
Cetuximab (+ PS80)	Untreated	(-)	(-)	(-)	(-)
Tocilizumab (+ PS80)	Untreated	(-)	(-)	(-)	(-)
Cetuximab (− PS80)	Silanized	(-)	N/A	(+ +)	(-)
Cetuximab (+ PS80)	Silanized	(-)	N/A	(+)	(-)

### Soluble Aggregate Analysis by SEC

#### Cetuximab and Tocilizumab without PS80 in Glass Vials

**Fig. 3:**
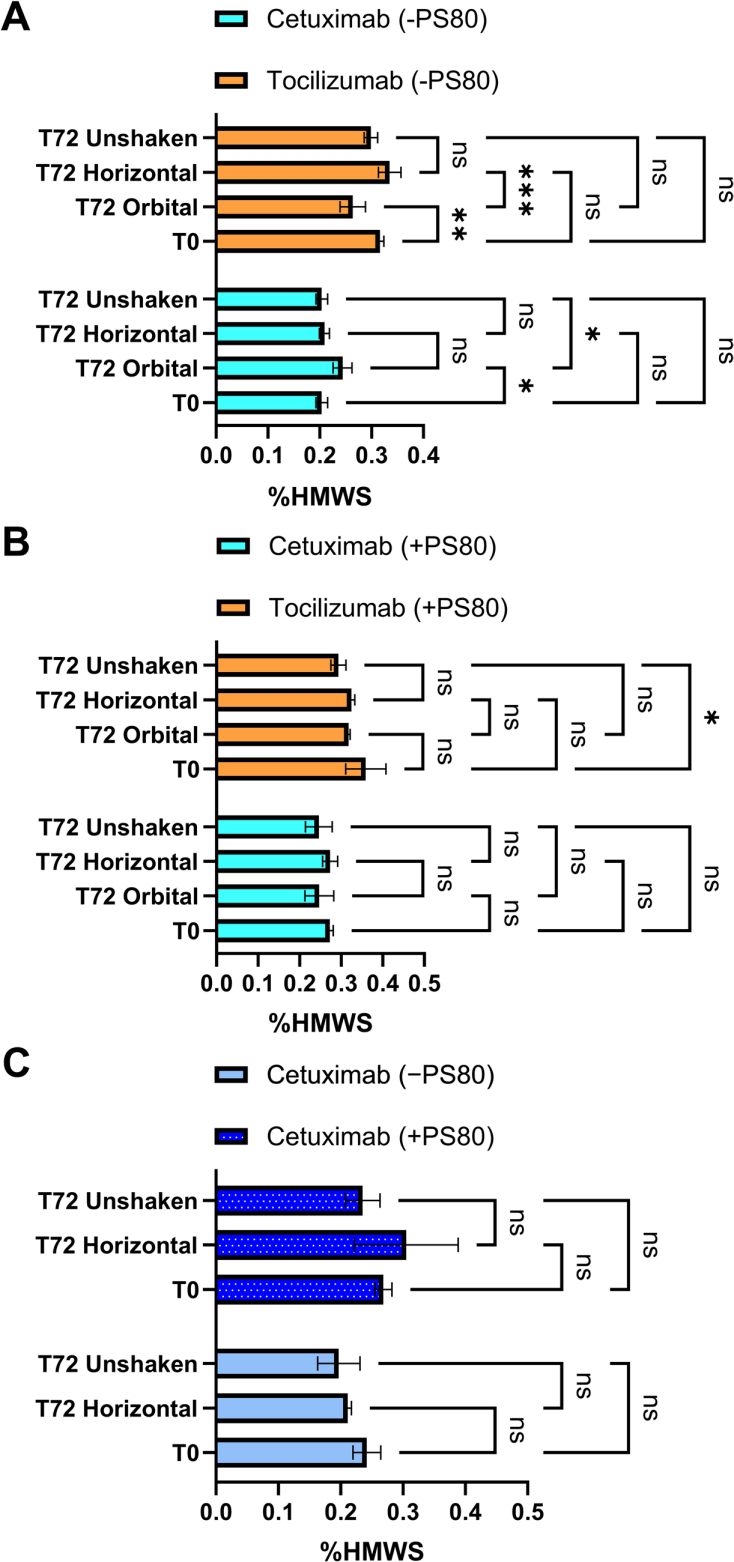
Percentage of high molecular weight species (%HMWS) in Cetuximab and Tocilizumab formulations under different shaking conditions, measured by size-exclusion chromatography (SEC). (**A**) mAbs without polysorbate 80 (PS80) in untreated glass vials, (**B**) mAbs with PS80 in untreated glass vials, (**C**) Cetuximab with and without PS80 in silanized glass vials

The %HMWS was assessed utilizing SEC to evaluate soluble aggregates post 72 h of shaking. Both Cetuximab and Tocilizumab were tested in untreated glass vials in the absence of the surfactant PS80. A smaller but statistically significant increase in %HMWS was observed only after orbital shaking relative to T0 (*p* = 0.0309) as well as T72 unshaken controls (*p* = 0.0309) for Cetuximab (Fig. 3A and Fig. S3A, B). For Tocilizumab, %HMWS was significantly lower after orbital shaking compared to both T0 (*p* = 0.0042) and horizontal shaking samples (*p* = 0.0002) (Fig. 3A and Fig. S3A, B). However, the absolute %HMWS values for both mAbs remained low (< 0.4%), along with some fluctuations or analytical variability, which limits the ability to draw any conclusions regarding %HMWS under these conditions and analyses alone.

#### Cetuximab and Tocilizumab with PS80 in Glass Vials

Upon the addition of 0.01% (w/v) PS80 to the mAb formulations, %HMWS generally stayed below 0.4% for both Cetuximab and Tocilizumab across all shaking conditions and timepoints. No statistically significant differences were observed between the control and post-agitation conditions for either mAb (p > 0.05) (Fig. 3B and Fig. S3A, B). A small but statistically significant difference was noted between T0 and T72 unshaken samples of Tocilizumab (*p* = 0.0315) (Fig. 3B and Fig. S3A, B). Since this difference arose between two unstressed conditions, both with low %HMWS, it does not reflect a meaningful stress-induced aggregation event and may instead result from integration variability or analytical noise.

#### Cetuximab with and without PS80 in Silanized Glass Vials

For exploring the influence of primary container surfaces, Cetuximab was also evaluated with and without PS80 in silanized glass vials. No statistically significant differences in %HMWS were observed across the conditions assessed, including T0, T72 unshaken, and T72 horizontal shaking samples (p > 0.05) (Fig. 3C and Fig. S3C). Overall, all the %HMWS remained consistently low (< 0.4%) and did not differ significantly for Cetuximab formulations both with and without PS80.

### Submicron Aggregate Analysis by DLS

#### Cetuximab and Tocilizumab without PS80 in Glass Vials

DLS was employed to monitor changes in hydrodynamic radius and PDI following agitation to evaluate the presence of early-stage submicron aggregates. Both Cetuximab and Tocilizumab formulations in untreated glass vials exhibited monodisperse size distributions across all timepoints, in the absence of PS80, with statistically insignificant changes observed (p > 0.05) (Table II). The hydrodynamic radius of Cetuximab remained consistent across T0, T72 unshaken, and T72 orbital shaking samples i.e., 5.5–5.7 nm, nonetheless an increase to 6.4 ± 1.4 nm following horizontal shaking was observed, accompanied by a multimodal PDI, indicating the onset of early aggregate species. In contrast, no multimodal peaks and stable size distributions were observed under all conditions for Tocilizumab (radius range: 5.4–5.6 nm, PDI: 0.0), signifying a greater resistance to agitation-induced submicron aggregation under surfactant-free conditions.

**Table II:** Hydrodynamic radius and polydispersity index (PDI) of Cetuximab and Tocilizumab formulations under different shaking conditions, measured by dynamic light scattering (DLS)

Monoclonal Antibody Formulation	Vial Type	DLS Parameter	T0	T72 Orbital	T72 Horizontal	T72 Unshaken
Cetuximab (− PS80)	Untreated	Radius (nm)	5.5 ± 0.0	5.5 ± 0.1	6.4 ± 1.4	5.7 ± 0.0
		PDI	0.0 ± 0.0	0.0 ± 0.0	Multimodal	0.0 ± 0.0
Tocilizumab (− PS80)	Untreated	Radius (nm)	5.5 ± 0.1	5.4 ± 0.1	5.6 ± 0.1	5.4 ± 0.1
		PDI	0.0 ± 0.0	0.0 ± 0.0	0.0 ± 0.0	0.0 ± 0.0
Cetuximab (+ PS80)	Untreated	Radius (nm)	5.5 ± 0.2	5.5 ± 0.1	5.5 ± 0.0	5.6 ± 0.1
		PDI	0.2 ± 0.2	0.0 ± 0.0	0.0 ± 0.0	0.0 ± 0.0
Tocilizumab (+ PS80)	Untreated	Radius (nm)	5.2 ± 0.0	5.3 ± 0.1	5.3 ± 0.1	5.3 ± 0.1
		PDI	0.0 ± 0.0	0.0 ± 0.0	0.0 ± 0.0	0.0 ± 0.0
Cetuximab (− PS80)	Silanized	Radius (nm)	5.6 ± 0.1	N/A	3.8 ± 2.8	5.9 ± 0.5
		PDI	0.0 ± 0.0	N/A	Multimodal	0.0 ± 0.0
Cetuximab (+ PS80)	Silanized	Radius (nm)	5.6 ± 0.1	N/A	5.6 ± 0.1	5.5 ± 0.1
		PDI	0.0 ± 0.0	N/A	0.1 ± 0.0	0.0 ± 0.0

#### Cetuximab and Tocilizumab with PS80 in Glass Vials

No statistically significant changes were observed for either mAb formulations containing PS80 (p > 0.05) (Table II). Specifically, Cetuximab showcased stable radii around 5.5–5.6 nm and consistently low, monodisperse distributions under all conditions. PS80 appeared to provide a stabilizing effect on Cetuximab under horizontal shaking stress with a measured radius of 5.5 ± 0.0 nm and PDI of 0.0 ± 0.0. For Tocilizumab in the presence of PS80, no measurable impact of shaking on the submicron particle profile was observed with consistent radii and PDI values.

#### Cetuximab with and without PS80 in Silanized Glass Vials

In both the Cetuximab formulations, with and without 0.01% (w/v) PS80, hydrodynamic radius values generally remained consistent across all conditions, (5.5 to 5.9 nm), with predominantly monodisperse PDI values without statistically significant differences (p > 0.05) (Table II). Interestingly, a deviation in radius and multimodal distribution was observed in the absence of PS80 following horizontal shaking, which supports that horizontal shaking is relatively more stressful for Cetuximab even in silanized glass vials. However, the PS80-containing samples showed a stable monomer radius without multimodal behavior, which further reinforces the role of surfactants in maintaining colloidal stability under mechanical stress.

### SVP Analysis by FIM

#### Cetuximab and Tocilizumab without PS80 in Glass Vials

**Fig. 4:**
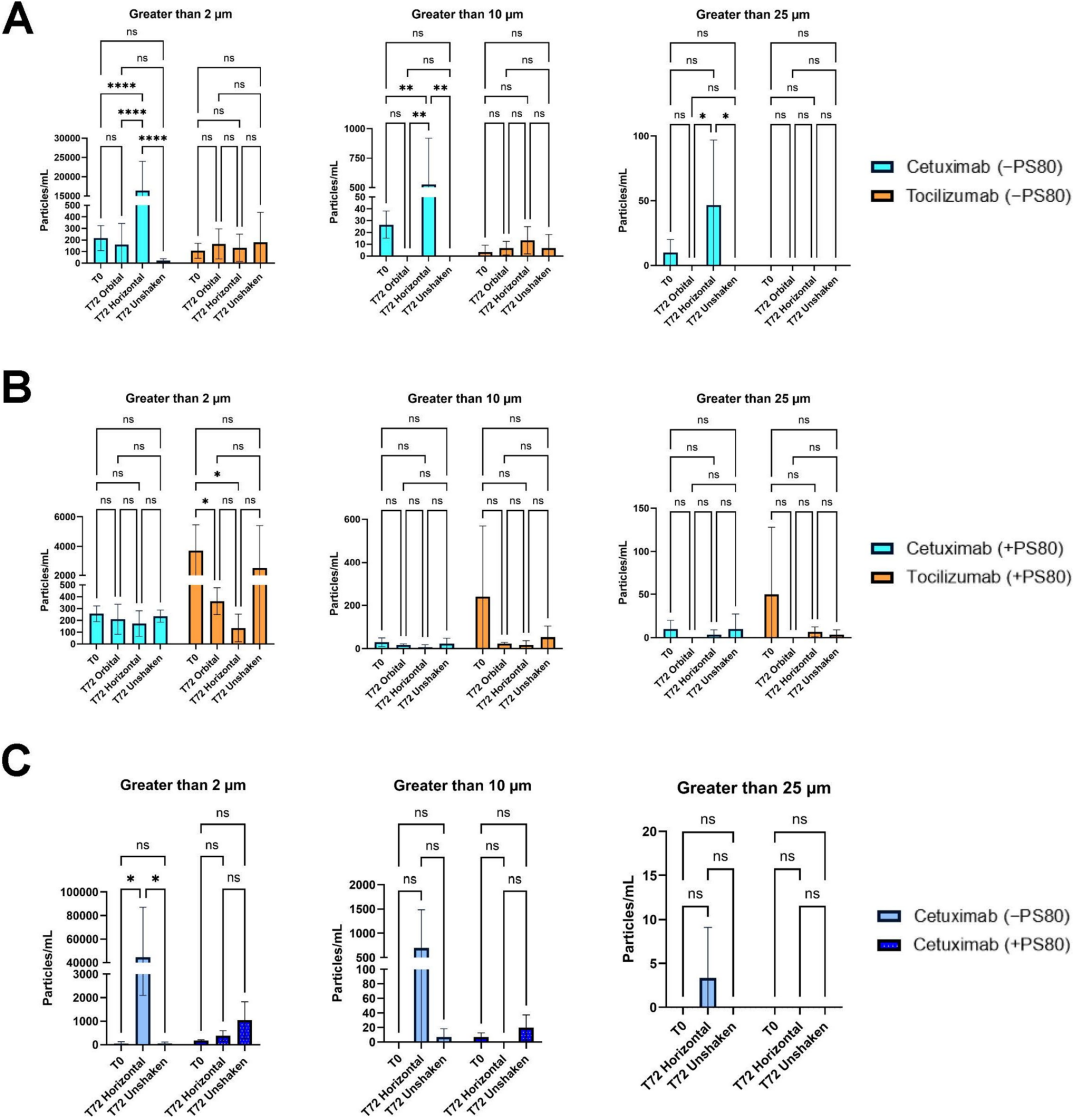
Subvisible particle (SVP) counts per mL in Cetuximab and Tocilizumab formulations under different shaking conditions, measured by flow imaging microscopy (FIM). Total particle counts are reported for three size thresholds: > 2 μm, > 10 μm, and > 25 μm. (**A**) mAbs without polysorbate 80 (PS80) in untreated glass vials, (**B**) mAbs with PS80 in untreated glass vials, (**C**) Cetuximab with and without PS80 in silanized glass vials

FIM was employed to quantify the SVP (> 2 μm, > 10 μm, and > 25 μm) under the influence of agitation. For Cetuximab, a significant increase in the concentration of particles was observed across all size thresholds with horizontal shaking in the absence of PS80 (Fig. 4A). Particularly, horizontal shaking induced a marked rise in greater than 2 μm particles (*p* < 0.0001, *p* < 0.0001, and *p* < 0.0001, respectively), and greater than 10 μm particles (*p* = 0.0023, *p* = 0.0014 and *p* = 0.0014, respectively), in comparison with T0, T72 orbital, and T72 unshaken conditions, respectively. For particles larger than 25 μm, statistically significant differences were observed between T72 horizontal shaking and both T72 orbital (*p* = 0.0285) and T72 unshaken samples (*p* = 0.0285). However, for Tocilizumab, the particle concentrations stayed statistically unchanged and low across all timepoints and stress conditions at each size range, suggesting minimal tendency for agitation-induced particle formation even without the surfactant (p > 0.05) (Fig. 4A). Overall, these data reveal a differential response to the type of agitation between the mAbs under surfactant-free conditions, with Cetuximab showing pronounced SVP generation upon horizontal shaking, and Tocilizumab showed relative resistance to shaking stresses, while both mAbs remained stable upon orbital shaking.

#### Cetuximab and Tocilizumab with PS80 in Glass Vials

For Cetuximab, the samples remained consistent across different timepoints and shaking stresses, with no statistically significant changes observed (p > 0.05) in the particles of all size ranges (Fig. 4B). However, Tocilizumab displayed significantly elevated counts in greater than 2 μm particles at T0 compared to T72 orbital (*p* = 0.0169) and T72 horizontal shaken samples (*p* = 0.0106) (Fig. 4B). Since no changes were observed for the post-agitation conditions, the differences were not stress-induced. While Tocilizumab appeared to have higher variability in greater than 5 μm and greater than 10 μm particles, the differences were not statistically significant (*p* > 0.05). Overall, the protective role of PS80 is evident for Cetuximab considering the lowered levels of SVP, whereas it may not be required for Tocilizumab under the shaking conditions.

#### Cetuximab with and without PS80 in Silanized Glass Vials

In silanized glass vials containing Cetuximab formulations without 0.01% (w/v) PS80, a statistically significant increase in particle counts greater than 2 μm was observed post horizontal shaking in comparison with both T0 (*p* = 0.0214) and T72 unshaken samples (*p* = 0.0214) (Fig. 4C). But no statistically significant differences were found in the greater than 10 μm and greater than 25 μm particle ranges (*p* > 0.05), in spite of higher particle counts post horizontal stress, and so the effect was more noticeable for smaller-sized SVP. For Cetuximab with PS80, no significant differences were observed in particle counts across any condition (*p* > 0.05) (Fig. 4C). Particle concentrations were consistently low, indicating that combining PS80 with silanized container surfaces essentially suppressed agitation-induced SVP formation. In contrast, the absence of PS80 still led to a significant increase in smaller SVP post horizontal shaking in silanized vials, an effect not seen when PS80 is present.

### Aggregate-Associated Scattering by UV–Vis Spectroscopy

#### Cetuximab and Tocilizumab without PS80 in Glass Vials

**Fig. 5:**
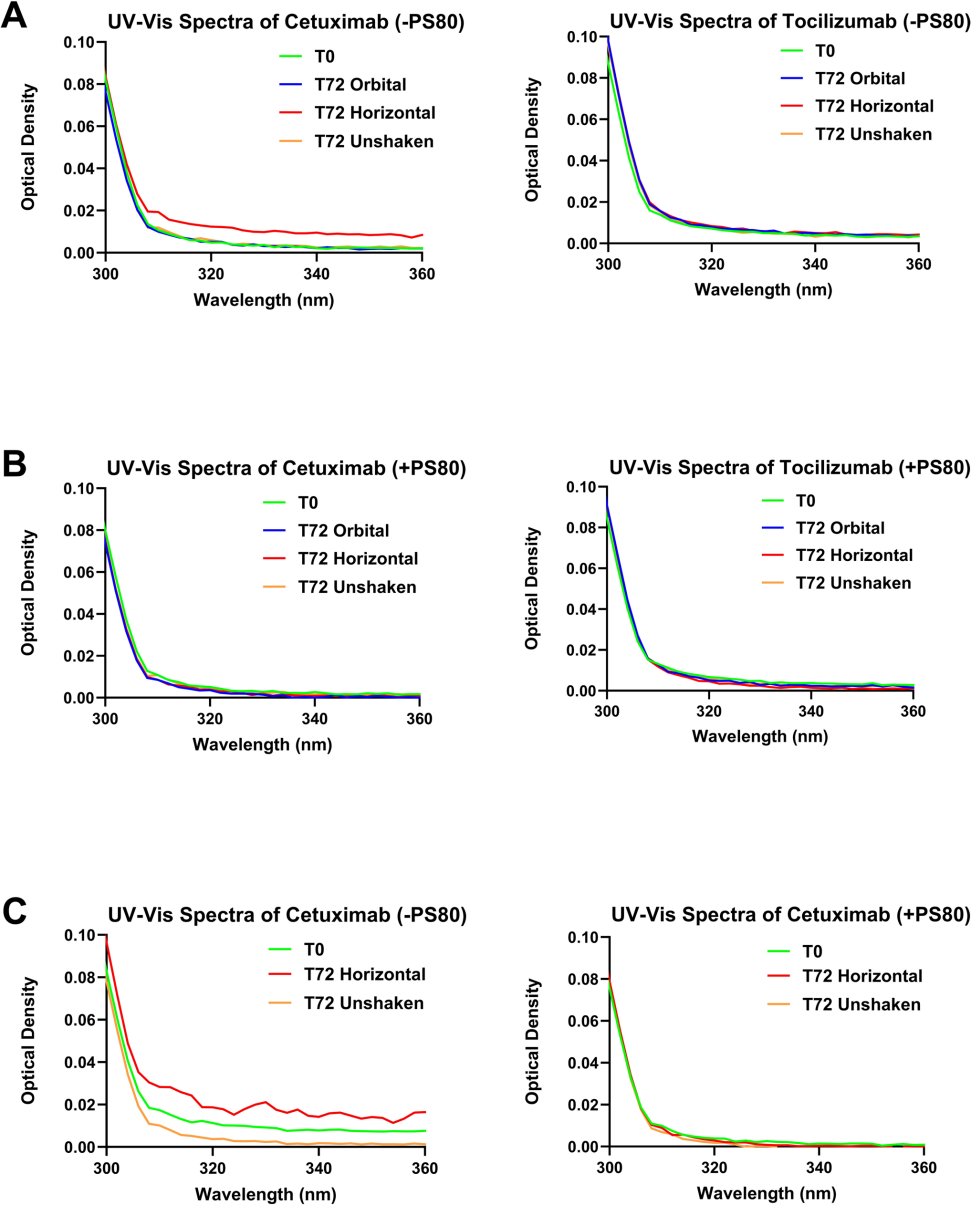
Ultraviolet–Visible (UV–Vis) spectroscopy results of Cetuximab and Tocilizumab formulations under different shaking conditions. The changes in optical density at 350 nm (OD_350_) were monitored as a proxy to assess aggregate-associated light scattering. (**A**) mAbs without polysorbate 80 (PS80) in untreated glass vials, (**B**) mAbs with PS80 in untreated glass vials, (**C**) Cetuximab with and without PS80 in silanized glass vials

UV–Vis OD_350_ was utilized as a proxy to assess aggregate-associated scattering. Across all the samples evaluated, Cetuximab in the absence of PS80 exhibited a clear increase in optical density post agitation. Particularly, horizontal shaking resulted in significantly increased optical density values in comparison with T0 (*p* < 0.0001), T72 unshaken (*p* < 0.0001), and T72 orbital samples (*p* < 0.0001) (Fig. 5A). These results are consistent with greater insoluble or submicron aggregate formation and in contrast, unshaken and orbital samples closely aligned with the T0 baseline. Besides, Tocilizumab without PS80 did not demonstrate significant changes across all the conditions which indicates decreased light scattering from aggregates or better stability (p > 0.05) (Fig. 5A).

#### Cetuximab and Tocilizumab with PS80 in Glass Vials

For both mAbs containing 0.01% (w/v) PS80 in untreated glass vials, the OD_350_ remained largely overlapping across all the conditions (T0, T72 Unshaken, T72 Orbital, T72 Horizontal). No statistically significant differences were observed between unstressed and stressed samples (p > 0.05) (Fig. 5B). The inclusion of PS80 for Cetuximab exhibited stable and low scattering profiles, even after horizontal agitation. Likewise, Tocilizumab samples maintained a similar baseline with no increases in agitation-induced aggregates via UV–Vis optical density (p > 0.05) (Fig. 5B). These findings imply that PS80 serves as a protective agent against UV-detectable aggregates in the mAbs using the tested shaking conditions.

#### Cetuximab with and without PS80 in Silanized Glass Vials

In silanized glass vials, Cetuximab without PS80 again demonstrated a statistically significant increase in OD_350_ post horizontal shaking as compared to the T72 unshaken samples (*p* = 0.0064) (Fig. 5C). This increase, consistent with elevated aggregate-associated scattering around 350 nm, an effect not observed in the controls. Contrastingly, Cetuximab containing PS80 displayed no significant differences in OD_350_ between any stress conditions and all spectra for the surfactant-containing samples remained smooth (p > 0.05) (Fig. 5C). This signifies that incorporating PS80 with the silanized container surface supports a stabilizing effect against aggregate formation.

## Discussion

### Phase 1: Cetuximab in Glass Vials

Visual inspection showcased the specific susceptibility of Cetuximab formulation without PS80, to aggregation, especially upon horizontal shaking. Cetuximab showed a relatively stable soluble aggregates profile across all tested conditions as assessed with SEC. While %HMWS for Cetuximab remained low across all stress conditions, aligning with Farjami *et al*., who also observed no significant SEC-detectable aggregation (recovery ~ 100%) after 48 h of shaking in room temperature at 150–250 RPM. These findings highlight an important caveat, as preserved monomer recovery may not necessarily guarantee preserved functionality, as they reported a marked reduction in receptor binding (from 97.6% to 67%) and diminished growth inhibition in A431 cells post-shaking [23]. Although the experimental setups differed from the current research (e.g., protein concentration, formulation, container type, shaking orientation), both studies reinforce the necessity of orthogonal or functional assessments to fully characterize stress responses.

DLS results pointed to relatively stable colloidal behavior in the nanometer size range for Cetuximab formulations, with early signs of aggregation only post horizontal shaking in the absence of PS80, despite the lack of statistical significance. The inclusion of PS80 mitigated these effects, underscoring its role in protecting against agitation-induced colloidal instability, via saturating interfaces, preventing protein–protein interactions, and aggregation upon unfolding events [8]. The sensitivity of Cetuximab to horizontal shaking stress was consistently evident across all SVP readouts from FIM size thresholds, with the most pronounced response observed for particles greater than 2 μm. Also, stabilizing role of PS80 for suppressing particle formation in agitation-sensitive Cetuximab, was further corroborated by SVP counts under horizontal shaking, with particles > 2 μm reducing from ~ 16,406/mL (without PS80) to ~ 173/mL (with PS80). In line with FIM trends, UV–Vis OD350 showed a clear and significant increase in light scattering for Cetuximab without PS80 subjected to horizontal shaking, suggesting insoluble aggregate formation, whereas PS80 addition prevented this rise across all conditions.

Overall, the findings for Cetuximab demonstrate that horizontal shaking is a more disruptive stress condition, capable of revealing aggregation sensitivity, which remains undetected under orbital shaking. This observation likely reflects the more aggressive, back-and-forth interfacial movement in horizontal shaking compared to the gentler, circular, and wall-hugging motion of orbital shaking [15]. Such distinctions underscore the risk of overestimating formulation robustness if only mild agitation modes are used and advocate the incorporation of horizontal shaking in stress testing panels to amplify interfacial perturbation and elicit otherwise latent aggregation tendencies. Notably, the addition of PS80 addressed the vulnerability of surfactant-free formulations to horizontal shaking, by protecting the air–liquid and solid–liquid interfaces [24], resulting in minimized interfacial film rupture and aggregate formation. Collectively, the results focus on the impact of selecting appropriate stress modalities, to ensure that forced degradation studies reflect formulation-relevant stress vulnerabilities.

### Phase 2: Tocilizumab in Glass Vials

Tocilizumab displayed robust stability across all analytical techniques, including minimal visual particles under both shaking conditions. As with Cetuximab, all %HMWS values for Tocilizumab remained low in absolute terms, suggesting a limited extent of soluble aggregates, reinforcing the need of interpreting these results with caution. DLS results indicate that Tocilizumab maintained colloidal stability under mechanical agitation, irrespective of the presence of the surfactant, highlighting its inherent robustness to shaking. Unlike Cetuximab, Tocilizumab showcased greater resistance to agitation-induced formation of SVP through different FIM size ranges across all shaking types and timepoints. Similarly, UV–Vis OD_350_ displayed minimum changes in Tocilizumab formulations regardless of PS80 presence through all the tested stress conditions.

In a side-by-side comparison with Cetuximab, these novel findings underline that horizontal shaking is not only a more aggressive stress condition but also a more discriminating tool in detecting formulation vulnerabilities. The contrasting stress responses of the two mAbs accentuates the ability of horizontal shaking to sensitively capture aggregation, proving the power of orthogonal stress testing in exposing molecule-specific risks. Additionally, the absence of measurable change upon adding PS80 to Tocilizumab suggests that its interfacial stability is adequate, and the surfactant may offer only marginal added benefit across all stresses. This superior robustness of Tocilizumab and its weak engagement at interfaces even without PS80, may be attributed to favorable molecular characteristics including molecular structure, surface properties or colloidal interactions, unlike Cetuximab. To further elucidate such molecule-specific behavior, a combination of predictive and in-silico tools, including CFD to simulate stress exposure at the interfaces and molecular modeling to characterize protein properties could offer mechanistic insights and guide formulation stress testing strategies [25–27]. Thus, these results indicate how stress sensitivity and excipient effects can be highly molecule-specific, emphasizing the importance of employing the appropriate excipient strategies and stress testing conditions during formulation development.

### Phase 3: Cetuximab in Silanized Glass Vials

Building on insights from earlier phases, Cetuximab was next utilized to assess how primary packaging surface chemistry, impacts aggregation under agitation. In silanized glass vials, Cetuximab without PS80 still formed visible particles, especially under horizontal shaking, despite the surface modification, hinting that silanization alone cannot fully mitigate interfacial stress [18]. No significant differences in %HMWS were observed upon shaking; nevertheless, DLS supported the presence of small, heterogeneous particles due to disruption of fragile protein films, which was not observed in Cetuximab with PS80. FIM and UV–Vis analyses further reinforced the complementary stabilizing effect on the mab via the combination of silanized glass surface and the PS80. Also, when comparing vial surfaces using formulations without PS80, FIM showed that silanization alone may aggravate aggregation in some cases, particularly under horizontal stress, with particles > 2 μm increasing from ~ 16,406/mL (in untreated glass) to ~ 44,593/mL (in silanized glass). Importantly, this was further verified by consistently low SVP counts observed in the associated buffer-only controls from FIM, confirming that the particles originated from mAb aggregation and not from interactions with the compatible vial coating (Fig. S4).

Generally, hydrophobic surfaces like silanized glass are known to promote increased protein adsorption [28], and interfacial adsorption-induced unfolding and conformational perturbation, can irreversibly initiate aggregation pathways under mechanical stress [29]. The modified wettability of silanized glass, especially in dynamic environments, likely affects meniscus movement and the behavior of the solid–liquid-air triple interface, a three-phase boundary critical in aggregation [30]. Although the air and liquid phases remain unchanged, increased air contact with silanized surfaces can promote greater dehydration and disruption of the adsorbed protein layer, facilitating its detachment into the bulk solution upon agitation. Then again, protein-to-protein and formulation-to-formulation differences can result in distinct stability profiles, as recently proven by Wang *et al*. They showed that shear-induced aggregate release from a glass surfaces can be lessened by octadecyltrichlorosilane (OTS) coating, which supports surface-anchored protein networks resistant to interfacial detachment. The hydrophobic coating (OTS-coated glass) ensured a reduction in SVP in comparison to untreated glass group after 14 days of static incubation [18]. Interestingly, in the current work, silanized glass exhibited greater interfacial instability without PS80 but showed comparably improved stability with PS80 across all analytical techniques. Thus, while hydrophobic silanized glass may offer a slight stabilizing edge under certain stress conditions, this effect appears to be surfactant-dependent, relying on effective interface saturation to prevent protein adsorption and gelation. The overall outcome is context-dependent, influenced by surface chemistry, excipient type and level, the degree of interfacial competition, as well as by molecular factors such as hydrophobicity, charge, and patchiness [31, 32]. This highlights the complementary relationship between surface-engineered vials and surfactant protection, focusing on the criticality of triple interface engineering to reshape interfacial dynamics and engineer robust biologics.

## Conclusion

In this work, Tocilizumab and Cetuximab formulations responded differently to agitation stress, vial surface properties and surfactant inclusion. Overall, horizontal shaking imposed greater stresses than orbital shaking, revealing aggregation in Cetuximab that would have been missed using orbital shaking alone. This study highlights the importance of selecting appropriate scale-down agitation models to capture the vulnerabilities of different molecules to mechanical and interfacial stresses. The addition of PS80 at 0.01% (w/v) effectively protected the mAbs against agitation-induced aggregation and ideally, excipient screening should go beyond standard surfactants to empower molecule-specific excipient compatibility assessments. Notably, silanized glass vials, when paired with PS80, reduced aggregation in Cetuximab formulations, by modulation of surface-protein interactions via altered interfacial energy landscapes, beyond simple adsorption phenomenon. Ultimately, a rational design of forced degradation studies along with molecule-specific, interface-aware, and translationally actionable drug product strategies are essential for a robust development campaign.

## Supplementary Information

Below is the link to the electronic supplementary material.

ESM 1 (DOCX 940 KB)

## Data Availability

Data will be made available on request.

## Abbreviations

ADCs: Antibody-drug conjugates
BsAbs: Bispecific monoclonal antibodies
CFD: Computational fluid dynamics
DDS: Dimethyldichlorosilane
DLS: Dynamic light scattering
FIM: Flow imaging microscopy
FPs: Fusion proteins
IgG: Immunoglobulin G
IV: Intravenous
mAbs: Monoclonal antibodies
OD_350_: Optical density at 350 nm
OTS: Octadecyltrichlorosilane
%HMWS: Percentage of high molecular weight species
PES: Polyethersulfone
PS80: Polysorbate 80
SEC: Size-exclusion chromatography
SVP: Subvisible particles
UV-Vis: Ultraviolet-visible
