# Supplementary material for: A Tale of Two Stressors in Biologic Drug Product Development: Shaking Mode and Primary Packaging
Source: Pharm Res. 2025 Nov 18;43(4):1305–18. doi: 10.1007/s11095-025-03959-4 (PMC13179228; doi:10.1007/s11095-025-03959-4)
Supplement: Supplementary file 1 — (DOCX 940 KB) [file 11095_2025_3959_MOESM1_ESM.docx]

**Supplementary Information**

**A Tale of Two Stressors in Biologic Drug Product Development: Shaking Mode and Primary Packaging**

Siddhanth Hejmady^a^, Elham Taherian^a^, Reza Nejadnik^a,^*

^a^ Department of Pharmaceutical Sciences & Experimental Therapeutics, College of Pharmacy, University of Iowa, Iowa City, IA 52242, USA

*Corresponding author:

Dr. Reza Nejadnik

Department of Pharmaceutical Sciences and Experimental Therapeutics

446 College of Pharmacy Building

180 S. Grand Ave

Iowa City, IA 52242, United States

E-mail: [reza-nejadnik@uiowa.edu](mailto:reza-nejadnik@uiowa.edu)

Website: <https://nejadnik.lab.uiowa.edu/>

**Figure captions:**

**Figure S1.** Representative photographs of vials vertically placed in tailor-made carton dividers, securely tightened to the two shaking platforms used in this study. Directional arrows illustrate the distinct motion patterns: circular for the orbital shaker (left) and back-and-forth for the horizontal shaker (right).

**Figure S2.** Representative photographs of formulations in untreated glass vials before and after shaking stress. Vials on the top show the Cetuximab formulation without polysorbate 80 (PS80) at T0, while vials on the bottom show the same formulation after 72 hours of horizontal shaking (n = 3).

**Figure S3.** Size-exclusion chromatograms (SEC) of Cetuximab and Tocilizumab formulations without PS80, under T0 and T72 horizontal shaking conditions. (A) Cetuximab in untreated glass vials, (B) Tocilizumab in untreated glass vials, (C) Cetuximab in silanized glass vials.

**Figure S4.** Subvisible particle (SVP) counts per mL in in phosphate-buffered saline (PBS), with and without PS80, in silanized glass vials under different shaking conditions, measured by flow imaging microscopy (FIM). Total particle counts are reported for three size thresholds: >2 µm, >10 µm, and >25 µm.


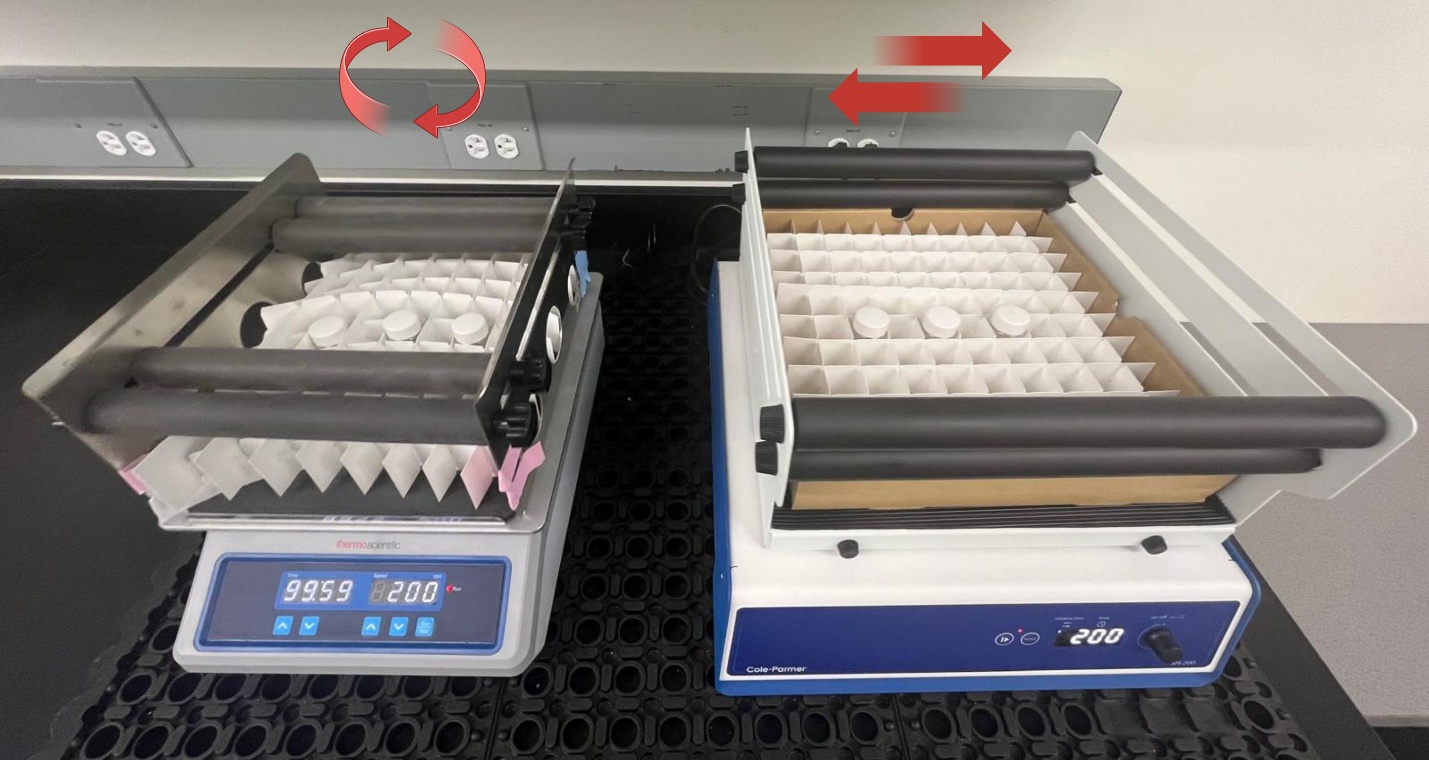
**Figure S1**


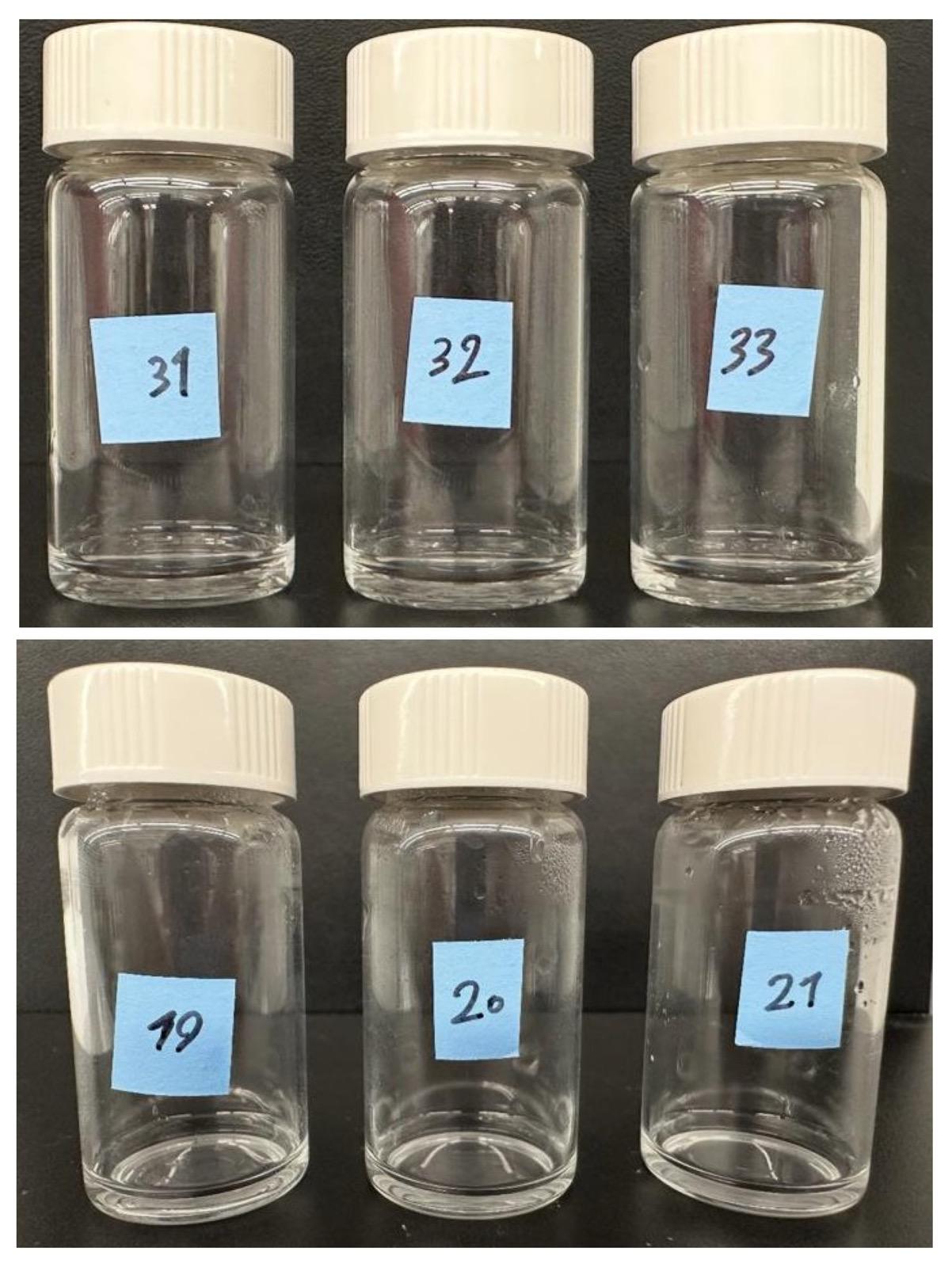


**Figure S2**


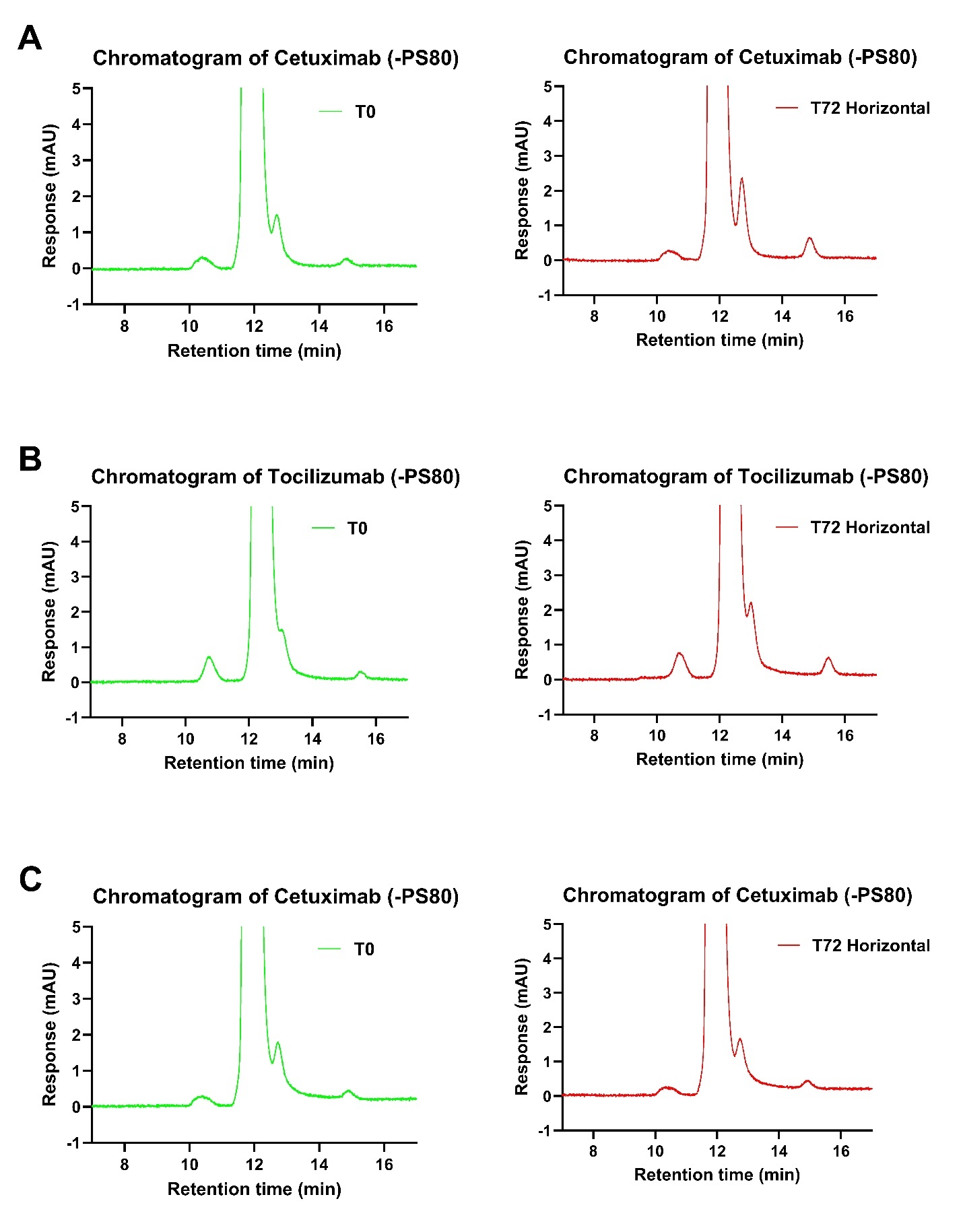
**Figure S3**

**
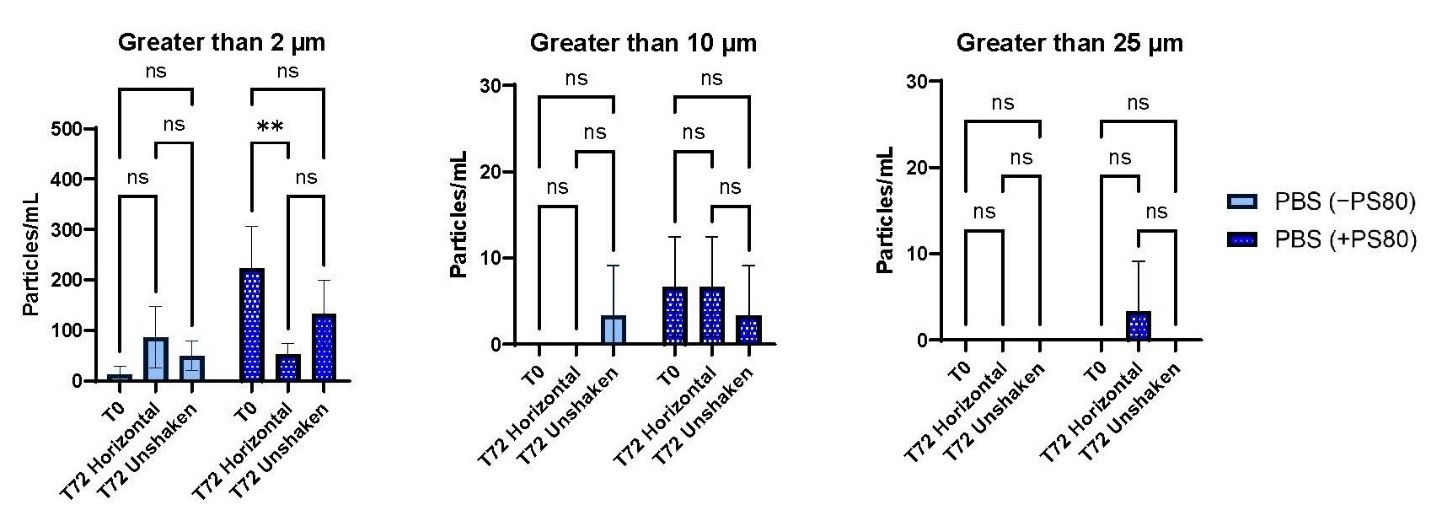
Figure S4**
